# Anti-angiogenesis in cancer therapeutics: the magic bullet

**DOI:** 10.1186/s43046-021-00072-6

**Published:** 2021-07-02

**Authors:** Ayodipupo S. Oguntade, Faez Al-Amodi, Abdullah Alrumayh, Muath Alobaida, Mwango Bwalya

**Affiliations:** 1 Nuffield Department of Population Health, University of Oxford, Oxford, UK; 2 Institute of Cardiovascular Science, University College London, London, UK; 3 Department of Basic Science, Prince Sultan Bin Abdulaziz College for Emergency Medical Services, King Saud University, Riyadh, Saudi Arabia

**Keywords:** Anti-angiogenesis, Vascular endothelial growth factor (VEGF), Antiangiogenics, Tumour resistance, Nanoparticle

## Abstract

**Background:**

Angiogenesis is the formation of new vascular networks from preexisting ones through the migration and proliferation of differentiated endothelial cells. Available evidence suggests that while antiangiogenic therapy could inhibit tumour growth, the response to these agents is not sustained. The aim of this paper was to review the evidence for anti-angiogenic therapy in cancer therapeutics and the mechanisms and management of tumour resistance to antiangiogenic agents. We also explored the latest advances and challenges in this field.

**Main body of the abstract:**

MEDLINE and EMBASE databases were searched for publications on antiangiogenic therapy in cancer therapeutics from 1990 to 2020. Vascular endothelial growth factor (VEGF) is the master effector of the angiogenic response in cancers. Anti-angiogenic agents targeting the VEGF and HIF-α pathways include monoclonal antibodies to VEGF (e.g. bevacizumab), small-molecule tyrosine kinase inhibitors (TKIs) e.g. sorafenib, decoy receptor or VEGF trap e.g. aflibercept and VEGFR2 inhibitors (e.g. ramucirumab). These classes of drugs are vascular targeting which in many ways are advantageous over tumour cell targeting drugs. Their use leads to a reduction in the tumour blood supply and growth of the tumour blood vessels. Tumour resistance and cardiovascular toxicity are important challenges which limit the efficacy and long-term use of anti-angiogenic agents in cancer therapeutics. Tumour resistance can be overcome by dual anti-angiogenic therapy or combination with conventional chemotherapy and immunotherapy. Emerging nanoparticle-based therapy which can silence the expression of HIF-α gene expression by antisense oligonucleotides or miRNAs has been developed. Effective delivery platforms are required for such therapy.

**Short conclusion:**

Clinical surveillance is important for the early detection of tumour resistance and treatment failure using reliable biomarkers. It is hoped that the recent interest in mesenchymal cell-based and exosome-based nanoparticle delivery platforms will improve the cellular delivery of newer anti-angiogenics in cancer therapeutics.

## Background

Cancers still account for significant morbidity and mortality globally despite remarkable advances in the management of cancers [1]. Cancers are characterised by alterations in vascular architecture and unregulated angiogenesis [2]. Angiogenesis is critical to tumour biology and continues to be the focus of research. Tumour hypoxia triggers the angiogenic switch via vascular endothelial growth factor and vascular endothelial growth factor receptor (VEGF/VEGFR) expression upregulation which drives tumour blood vessels viability and tumour cell survival. Kim et al. [3] are perhaps the first pre-clinical model of antiangiogenic therapy demonstrated that a monoclonal antibody against VEGF inhibited tumour growth in mice injected with human cancer cell lines. This led to the development of drugs targeting different points of the VEGF/VEGFR signaling cascade. Bevacizumab, a monoclonal antibody to VEGF, was the first anti-angiogenic to be approved for the treatment of cancers [4]. It is now approved in combination with chemotherapy for metastatic colorectal cancer and also used in other advanced malignancies [5, 6]. Since its development, several molecules have been synthesized and approved for the treatment of different solid organ cancers [7, 8]. Despite the initial success with the use of these agents, they are associated with eventual treatment resistance and cardiotoxicity. The identification of reliable biomarkers of treatment response and the use of these antiangiogenic agents with conventional chemotherapy and immunotherapy have prospects to improve the care of individuals with cancers.

The main aim of this review is to discuss the different anti-angiogenic agents in cancer therapeutics and the mechanisms and management of tumour resistance to antiangiogenic agents. We also reviewed the use of combination therapy in overcoming resistance to antiangiogenic therapy and the significance of their cardiotoxicity in clinical care. The advances in the use of nanoparticles and tumour stem cells as antiangiogenic therapy are also discussed.

## Main text

We searched MEDLINE and EMBASE for publications on anti-angiogenesis in cancer from 1990 to 2020 as part of a larger project on anti-angiogenesis and cancer therapeutics. Keywords associated with ‘cancer’, ‘malignancy’, ‘tumour’, ‘neoplasm’, ‘anti-angiogenesis’, ‘angiogenesis inhibitor’, ‘vascular endothelial growth factor’, ‘treatment outcome’, ‘tumour resistance’, ‘antiangiogenic drug cardiotoxicity’ and ‘anti-angiogenic nanoparticle’ were used for the search. The search was limited to articles published in the English language.

### Anti-angiogenics in cancers

Several preclinical and clinical studies in cancer research have targeted different steps of the angiogenic pathway. Among these targets are growth factors and their receptors, which are released by tumour or stromal cells, such as VEGF, angiopoietin, hepatocyte growth factor (HGF), platelet-derived growth factor (PDGF), VEGFR, placental growth factor and its receptor (PIGF/PIGFR), fibroblast growth factor and its receptor (FGF/FGFR), Tei receptors and PDGF receptors. In addition, tyrosine kinase receptor activity and the hypoxia-inducible factor-1α (HIF-1α) system have been studied as targets for anti-angiogenic drugs.

Anti-angiogenic agents targeting the VEGF pathway include monoclonal antibodies to VEGF (e.g. bevacizumab), small-molecule tyrosine kinase inhibitors—TKIs (e.g. sorafenib), decoy receptor or VEGF trap (e.g. aflibercept) and VEGFR2 inhibitors (e.g. ramucirumab). These classes of drugs are vascular targeting which in many ways are advantageous over tumour cell targeting drugs [9].

Monoclonal antibodies are the most accepted class of drugs in therapeutic anti-angiogenesis, one of which is Bevacizumab. It mainly acts by binding to circulating VEGF which in turn inhibits its binding to cell surface receptors [10]. This leads to a reduction in the tumour blood supply and a reduction in the growth of the tumour blood vessels [10]. Bevacizumab (Avastin), a humanized anti-VEGFA monoclonal antibody in combination with IFL (irinotecan, 5FU and leucovorin), was approved for the treatment of metastatic colorectal carcinoma by the US Food and Drug Administration (FDA) in February 2004 [11]. The E2100 trial of bevacizumab plus paclitaxel in breast cancer also showed benefit leading to its approval in metastatic breast cancer in 2008 [12]. However, the AVADO [13] and RIBBON-1 [14] trials even though, showed improvement of progression-free survival with bevacizumab use, did not show any benefit of overall survival. This led to its withdrawal in metastatic breast cancer by the FDA in 2011.

Aflibercept is a fusion protein composed of the constant Fc domain of human IgG combined with the second immunoglobulin domain of VEGFR-1 and the third immunoglobulin domain of VEGFR-2. It acts like a VEGF trap and a decoy receptor of angiogenic factors. It targets VEGFA, VEGFB and PIGF. It is used for the treatment of metastatic colorectal cancer. In the VELOUR phase II trial of patients with advanced colorectal cancer who had failed an oxaliplatin-based regimen, patients on aflibercept showed significant improvement in overall survival and progression-free survival [15]. However, in the VITAL study, a phase III trial of aflibercept plus docetaxel vs. docetaxel alone in patients with advanced non-small-cell lung cancers (NSCLC) who had failed therapy with a platinum-based regimen, aflibercept did not affect overall survival though it reduced progression-free survival [16].

Ramucirumab is a human monoclonal antibody that blocks the interaction between VEGF and its receptor by binding to the extracellular domain of VEGFR2. It has high selectivity for VEGFR2. Following the RAISE study, it was approved in combination with folinic acid, 5-fluorouracil and irinotecan for the treatment of metastatic colorectal cancers that have progressed despite therapy with bevacizumab, oxaliplatin and fluoropyrimidine [17]. It is also approved as second-line therapy for gastric and NSCLC [18].

The TKIs are tyrosine kinase/serine/threonine kinase or dual protein kinase inhibitors. Some target VEGFRs (e.g. sunitinib and sorafenib) but they often target other pathways (e.g. PDGFR, FGFR and c-Kit). Details of their action are shown in Table 1. These medications are susceptible to resistance when used as monotherapy. There is also concern that they may increase the malignant potential of cancer cells.

**Table 1:** Selected VEGF-targeted anti-angiogenics and their therapeutic indications

Generic name	Mechanism of action	Indication
Bevacizumab	Humanised VEGF monoclonal antibody	Colorectal cancer (CRC), non-small cell lung cancer (NSCLC), metastatic breast cancer, glioblastoma, metastatic renal cell cancer (mRCC)
Sorafenib	Tyrosine kinase inhibitor (TKI)—VEGFR1, VEGFR2, VEGFR3, PDGFR, Raf kinase, FGFR, c-fms	mRCC, metastatic hepatocellular cancer (HCC), thyroid cancer
Sunitinib	As for sorafenib, also Kit, FLT3, CSF-1R, RET	Advanced RCC, HCC, gastrointestinal stromal tumours (GIST), advanced pancreatic cancer, and neuro-endocrine tumours
Vandetanib	EGFR, VEGFR2, VEGFR3, RET	Unresectable or metastatic medullary thyroid cancer
Axitinib	TKI (VEGFR1, VEGFR2, VEGFR3, PDGFR, c-Kit)	Advanced RCC, pancreatic cancer
Pazopanib	TKI (VEGFR1-3, PDGFR, c-Kit, Itk, LcK, c-FMS), ABL-1,	mRCC, advanced soft tissue sarcoma
Regorafenib	TKI (VEGFR2-3, PDGFRβ, Raf, Ret, c-Kit, Tie2)	Unresectable GIST, mCRC, HCC
Ramucirumab	Monoclonal antibody that binds extracellular domain of VEGFR2	Metastatic NSCLC, metastatic gastric and CRC
Aflibercept	Receptor fusion protein (VEGFA/ VEGFB trap, active against PIGF	CRC, NSCLC, prostate cancer
Cetuximab	TKI (EGFR)	CRC, head and neck cancer

### Dll4 and notch inhibitors

Dll4 and Notch are upregulated by VEGFA and act as negative feedback for vessel sprouting and angiogenesis under normal physiologic conditions. When Dll4 downregulation with siRNA was combined with anti-VEGF therapy, it resulted in greater tumour growth inhibition than either alone [19]. MEDI0639, a Dll4-Notch disrupter has shown promise in a preclinical study [19]. Demcizumab, another Dll4 inhibitor, has been trialed in pancreatic, metastatic colorectal cancers and NSCLCs [20].

### HIF-1α system inhibitors

After discovering the role of HIF system in the expression of different genes and proteins that are essential for tumour growth and survival, this system has become a target for newly investigated tumour therapeutics [21].

Agents have been discovered that inhibit different steps of HIF1-α signaling, from its expression to DNA binding and transcription. Jeong et al. [22] have developed EZN-2968, an antisense oligodeoxynucleotide that binds to a complementary sequence in the mRNA of human HIF1-α and downregulates it. A phase I trial has evaluated this molecule and found that the expression of HIF1-α was reduced in four out of six patients with solid tumors [22]. Despite tremendous research in this area, no drug directly tackling this system has been approved for cancer therapy yet. This remains a promising therapeutic area.

### Angiopoietin-Tie2 axis inhibitors

The angiopoietin-Tie axis is another important pathway in tumour angiogenesis. Both Ang1 and Ang2 are upregulated in many tumours, but each has a different effect on Tie2 signaling. Ang1 binds to Tie2 receptor causing a reduction in vascular permeability and promotion of vessel maturation and stabilization. Ang2 antagonises Ang1 and induces neovascularization by destabilizing endothelial-pericyte junctions and promotes endothelial cells (EC) survival, migration and proliferation. Thus, a higher ratio of Ang2 to Ang1 levels predicts worse clinical outcomes. The effect of Ang2 signaling appears to largely depend on other proangiogenic cytokines being present e.g. VEGFA.

Ectopic Ang2 expression interferes with VEGFR2 blockade and combined inhibition of Ang2 and VEGFA produce a greater reduction in angiogenesis in laboratory models. Regorafenib, a multi-target RTK inhibitor with VEGFR1-3 and Tie2 activity, demonstrated efficacy as third-line therapy for metastatic colorectal cancer and gastrointestinal stromal tumours (GIST) [20]. Trebananib is a peptide Fc fusion protein that inhibits the interaction between Ang1, Ang2 and Tie2. It has shown promise in phase II trials. It has been combined with paclitaxel, carboplatin and liposomal doxorubicin in phase III trials [23].

A summary of anti-angiogenics in clinical use is shown in Table 1. These antiangiogenics inhibit tumour growth by blocking vascular supply, triggering degeneration of vascular networks, cellular apoptosis, stimulating tumour hypoxic death and modulating inflammatory cells and effectors.

Contrary to the initial hope about anti-angiogenics in cancer therapy, these agents only increase survival by an average of few months. Furthermore, the failure to identify and validate durable predictive markers of response, and the need to better characterize the mechanisms of tumour resistance have been the challenges limiting anti-angiogenic therapy. Even though inhibition of VEGF pathways has anti-tumour effects in mouse cancer models, they elicit tumour adaptation, increased invasiveness and metastasis through the upregulation of alternative growth and angiogenic pathways [24].

### Tumour resistance to anti-angiogenic therapy

Many patients treated with VEGF inhibitors especially when combined with chemotherapy may survive longer, but they eventually succumb to their disease. VEGFA may be replaced by other angiogenic pathways as the disease progresses. These include VEGF upregulated pathways and other pathways mediated by other members of the VEGF family which may bind to and activate VEGFR2 after proteolytic cleavage. Investigators have identified other mechanisms of failure and resistance to anti-VEGF therapy. The hypoxic environment of tumours while on anti-VEGF therapy results in upregulation of other chemokines and growth factors e.g. bFGF, PDGF, HGF, IL-1, IL-8 and ephrins which become hypoxia independent and do not respond to bevacizumab [25, 26]. This facilitates rebound angiogenesis, tumour revascularization, escape from immune cells and tumour invasion [24]. This has been shown in patients with colorectal cancers and renal cell cancers. Moreover, hypoxia after tumour regression following VEGF blockade can lead to a switch to a more invasive nature since in some cases, cancer stem cells can become tolerant to hypoxia following the acquisition of extra mutation. In addition, VEGF blockade may not be effective in suppressing other pathways of vascularization especially those that rely on recruitment of bone marrow-derived cells, vascular mimicry or vessel co-option. Some tumours are also largely hypovascular e.g. pancreatic cancer and may not respond to anti-VEGF therapy. Furthermore, tumour vessel remodeling results in a shift to mature stabilized vessels that are less responsive to antiangiogenic therapy.

It appears that signals from the stromal component of tumours play a role in acquired resistance to antiangiogenic therapy. Cells of the bone marrow origin especially endothelial progenitor cells (EPCs) and CD11b+Gr1+ cells are implicated in metastasis [27]. Bone marrow VEGFR+ cells are also implicated in the formation of a pre-metastatic niche before the arrival of tumour cells. Moreover, EPCs are involved in the angiogenic switch from micro-metastasis to macro-metastasis. These cells are recruited into premetastatic sites in response to SDF-1 and CXCL15 gradients and promote metastasis via metalloproteinase-induced pathways [27]. Thus, targeting myeloid cells and their homing into tumour sites may break the jinx. This behaviour of tissue EPCs and myeloid cells can be used as predictive markers of response to antiangiogenic therapy as discussed later below.

Endothelial to mesenchymal transition in cancer cells contributes to increased angiogenesis, invasiveness and unresponsiveness to VEGF blockade. Cancer-associated fibroblasts contribute to tumour angiogenesis via the release of stromal-derived factor (SDF-1) which leads to the recruitment of bone marrow cells and assembly of the endothelial population in the tumour vasculature [28]. This occurs via hypoxia-induced HIF-1α activation. SDF-1 can stimulate CXCR7 leading to proangiogenic cytokine secretion by endothelial progenitor cells. Such CXCR7/SDF-1 signaling is involved in the migration and homing of angiogenic and immune cells to areas of tumour growth [11]. The recruitment of myeloid-derived suppressor cells leads to a weakened antitumour response. Myeloid cells of the mononuclear macrophage lineage are activated and mediate multiple pathways that lead to tumour progression and angiogenesis. Also, there is selection pressure that leads to overgrowth of tumour cell variants that are resistant to hypoxia-mediated angiogenesis. It may also be that doses of current anti-VEGF therapies are not optimal for targeting cure. Furthermore, integrin-mediated signaling in vascular beds may provide alternative mitogenic and survival signals. Evidence from preclinical studies has shown the interaction between integrins and receptor tyrosine kinases (RTKs) in tumour invasion [29]. Genetic alterations in tumours may decrease the vascular dependence of tumour cells and affect therapeutic response to antiangiogenic therapy. In the study by Yu et al., mice with p53 knock-out mutations were less responsive to antiangiogenic therapy than mice with wild-type p53 tumours [30].

Vessel co-option is another mechanism of tumour resistance to anti-angiogenic therapy [26]. Tumour cells can incorporate existing vasculature to accelerate their growth. This has been shown in gliomas and lung cancers and in patients with colorectal cancer treated with bevacizumab [31]. Tumour cells also use vasculogenic mimicry to evade antiangiogenic therapy. They can differentiate and gain EC-like features e.g. expression of VE cadherin and ephrin A2. This is important for invasion and metastasis.

### Anti-angiogenics in clinical care: combination therapy to overcome tumour resistance

An interesting concept in anti-angiogenic therapy is vascular normalization and re-distribution of flow in tumour vascular bed when anti-angiogenics are combined with the conventional chemotherapy regimen [32]. It has been suggested that normalising the tumour vasculature would diminish endothelial and perivascular cells, decrease the high interstitial pressures in solid tumours, enhance oxygenation and chemotherapy delivery into tumour cells [11]. Antiangiogenic agents do not achieve enough efficacy when they destroy tumour vascular networks as monotherapy but rather, by pruning tumour vascular networks when administered with other chemotherapeutics, they reduce vascular hydrostatic pressure, tumour-associated oedema and temporarily improve tumour hypoxia, thus improving delivery and activity of chemotherapeutics which can then effectively destroy tumour cells. This has been demonstrated in colorectal cancers and glioblastoma multiforme [32, 33]. Recently, a combination of bevacizumab with paclitaxel and carboplatin in patients with non-small cell lung cancer (NSCLC) has also shown improved survival [11].

In tumours, molecules involved in immune checkpoint e.g. programmed death 1 (PD-1) expressed on natural killer T cells, CD8^+^T cells, B cells and antigen-presenting cells (APCs) are highly expressed. PD-1 interacts with its ligand, PD-L1 in immune and cancer stromal cells to inhibit the proliferation and survival of T cells which are important in immune surveillance of tumours [33]. Hijacking of PD-PD-L1 pathway activation by solid tumours leads to T cell exhaustion and increased expression of FoxP3 by regulatory T cells (Tregs) with resultant immunosuppression and tumour resistance. Tregs also constitutively express CTLA4 which has strong immunosuppressive effects via the downregulation of CD80 and CD86 on antigen-presenting cells (APCs) and inhibition of CD8^+^ effector T cells. Immunotherapy with PD/PD-L1 blockage (pembrolizumab, atezolizumab, nivolumab) or CTLA4 blockade (ipilimumab) increases survival in metastatic solid tumours like NSCLCs and renal cell cancers. Interestingly, potent antiangiogenic therapy promotes tumour hypoxia and upregulation of the PD/PD-L1 pathway with consequent immune suppression while low-dose VEGFR2 blockade increased tumour vascular infiltration with CD8^+^effector T cells. The combination of low-dose VEGFR2 blockade and a cancer vaccine also led to an increased immune response to tumour cells, vascular normalisation and improved survival in mice models of breast cancer and colon cancer [34, 35]. There are now ongoing trials investigating the role of dual anti-angiogenic therapy and immunotherapy (using bevacizumab with atezolizumab) e.g. in advanced renal cell cancers (NCT02420821) [33]. Triple therapy using a combination of anti-angiogenic agents, immunotherapy and conventional chemotherapy are also being trialed in metastatic solid tumours (NCT02839707, NCT02366143) [33]. These trials have a high potential for overcoming of tumour resistance to anti-angiogenic molecules in future.

### Biomarkers of tumour response to anti-angiogenic therapy

Reliable biomarkers of tumour response to antiangiogenic therapy have become a focus of attention given the risk of tumour resistance and adverse events. However, most of the studies have been inconsistent. Circulating VEGF levels have been investigated as a predictive biomarker of response to anti-VEGF therapy. In the study by Hillan et al. [36], which evaluated the role of VEGF expression in response to bevacizumab plus capecitabine in metastatic breast cancer, the response rate was not different in those on bevacizumab who had VEGF overexpression compared to those without VEGF overexpression. In the TARGET trial which investigated sorafenib in advanced renal cell carcinoma, serum VEGF levels had an inverse relationship with progression-free survival and overall survival [37]. Taken together, it seems that while VEGF has prognostic value, it is not a reliable predictor of response to therapy.

Vascular endothelial cadherin is another potential biomarker [38]. It is important in maintaining EC contact. It also plays important role in regulating cell proliferation, apoptosis and modulates VEGFR2 function. In the same vein, integrins that mediate cell-cell and cell-extracellular matrix interactions may be important biomarkers because of their roles in tumour invasion and metastasis. Nanoparticles bearing αvβ3 integrins are being investigated for molecular tumour imaging. Other possible biomarkers of response include plasma levels of growth factors, and tumour expression of VEGF/VEGFR and other signaling pathways [39, 40]. Circulating levels of HGF, IL-6, IL-8, osteopontin and TIMP1 have been shown to identify patients who had greater overall survival benefit from treatment in pazopanib-treated patients with metastatic renal cell cancers in one study [41]. Challenges with the use of circulating biomarkers include the absence of standardization of measurements across centres and the absence of accepted cut-off levels for these circulating biomarkers. Moreover, circulating factors tend to fluctuate in disease settings and disease stage.

Mast cells and miRNAs are increasingly being investigated as diagnostic and prognostic biomarkers in tumours like colorectal cancers and are potential therapeutic targets [42]. High mast cells density is correlated with the advanced stage of colorectal cancer and tumour progression. Recently, mast cell tryptase inhibitors e.g. gabexalate mesylate and nafamostat mesylate have been studied in metastatic gastric cancers with encouraging result [43]. There has been an interest in non-coding miRNAs in colorectal cancer progression. miRNA-21 and miRNA-320 are oncogenic miRNAs seen at all stages of colorectal cancer progression [42]. Their levels in tumour tissues have been correlated with survival in individuals with colorectal cancers. miRNA-21 has been shown to confer tumour resistance to 5-fluoro uracil by downregulating MutS homologue-2 while high levels of miRNA-203 have been correlated with oxaliplatin resistance [42]. The development of drugs which target the secretion or action of these miRNAs holds great promise for the prevention and treatment of tumour resistance in patients on anti-angiogenic treatment and conventional chemotherapy.

Microvascular density in serial tumour biopsies has been proposed as a reliable biomarker of response along with the measurement of circulating angiogenic markers and adhesion molecules [44]. A meta-analysis showed that micro-vessel density predicted survival in non-small cell lung cancer (NSCLC) [45]. However, in a study of colorectal patients treated with chemotherapy with/without bevacizumab, pretreatment micro-vessel density was not a significant predictor of the benefit of bevacizumab addition to treatment [45]. Anti-angiogenics may not only affect tumour vessels but also the normal vasculature; thus, healthy tissue in tumours may be used to monitor antiangiogenic therapy in tumours. Vessel density and intra-tumour blood supply may be estimated using imaging methods like contrast-enhanced MRI or PET. In one clinical trial of metastatic colon cancer, epithelial and stromal VEGF expression and micro-vessel density were not predictive of the benefit of the addition of bevacizumab to 5-fluorouracil based therapy [46].

Vascular imaging using ultrasound, CT, MRI or PET is another predictive marker that can be used to assess response to treatment as shown by the use of MRI in monitoring response to antiangiogenic therapy in patients with glioblastoma multiforme (GBM) [47]. High levels of vascular perfusion on vascular imaging predicted response and outcome in patients with metastatic renal cell cancers who were treated with TKIs [48]. A recent study by Rojas et al. [49] suggested that ultrasound molecular imaging may also be a better marker of response to therapy. Challenges with using these imaging modalities include marked variability in methodologies used to assess imaging biomarkers across studies and the need for standardization of tumour molecular imaging. Different types of biomarkers (e.g. circulating and imaging) may have to be combined to yield a composite biomarker for more robust predictors of response to antiangiogenic therapy.

### Cardiovascular effects of anti-angiogenic therapy

The cardiovascular adverse effects of antiangiogenic therapy are worthy of mention. Some of the reported side effects are hypertension, cardiac dysfunction and myocardial ischaemia.

These agents act by reducing nitric oxide expression which leads to vasoconstriction and elevation of blood pressure [50]. Other pathophysiologic pathways for hypertension include increased expression of endothelin-1, microvascular rarefaction, activation of the renin-angiotensin-aldosterone axis, oxidative stress, pressure natriuresis and arterial stiffness.

VEGF signaling pathway inhibitors cause an increase in blood pressure with 7.4% developing severe hypertension in a meta-analysis with a number need to harm of 6 for hypertension [50]. Blood pressure elevation occurs rapidly within hours or days of starting anti-VEGF therapy and is commensurate with effective VEGF signaling inhibition. It remains unclear whether blood pressure goals in such patients should be the same as for the general population even though current hypertension guidelines do not discriminate between these patients and the general population. The risk of hypertensive target organ damage is increased in these patients. The National Cancer Institute recommends formal cardiovascular assessment before commencing anti-angiogenic therapy, and antihypertensives should be commenced in such patients once there is a more than 20mmHg rise in diastolic blood pressure from baseline even if blood pressure remains in the normotensive range [51]. There is a need to clarify the blood pressure threshold at which anti-angiogenic dose reduction or termination should be considered. The preferred classes of antihypertensives in such instances are also a matter of debate. It is better to avoid non-dihydropyridine calcium channel blockers since they inhibit the CYP3A4 which is responsible for the metabolism of antiangiogenic medications and can thus elevate plasma levels of anti-angiogenics with resultant worsening of hypertension.

Anti-angiogenic therapy has been implicated in cardiotoxicity. The risk is particularly high in those who develop hypertension. Moreover, the risk of left ventricular (LV) dysfunction remains high among patients whose blood pressure has been controlled while on medications like sunitinib. It appears that chronic afterload increase leads to the hypertrophic myocardial response, increased oxygen demand and activation of the HIF1-α/VEGF axis that leads to vascular neogenesis [50]. Such capillary density may not match the increase in myocardial area or hypertrophy. This mismatch causes reduced fractional shortening and increased LV end-diastolic pressure [50].

In mice treated with TKIs like sunitinib and also in patients on anti-angiogenic therapy, there is capillary rarefaction and myocyte mitochondrial swelling and degenerative changes which are compounded by apoptosis in those with high blood pressure [50]. It appears that increased afterload accelerates this capillary rarefaction and may underlie the development of LV dysfunction. Cardiotoxicity also involves alteration in myocardial energetics via AMP-kinase inhibition and resultant mitochondrial dysfunction. Such changes lead to reduced contractility and increase the susceptibility of the heart to other insults. Such cardiotoxicity may be due to both on-target and off-target effects of TKIs on the heart which leads to adverse remodeling and cardiac dilatation. This underscores the need to monitor left ventricular function in patients on anti-angiogenic therapy.

Myocardial ischaemia has been observed with some antiangiogenic agents including bevacizumab, sunitinib, sorafenib and regorafenib [50]. This LV dysfunction is usually asymptomatic and is reversible on early withdrawal of such therapy. Risk factors for such arterial thrombotic events are unclear but background heart disease, hypertension, older age and use of other cardiotoxic drugs likely play important roles.

**Fig. 1:**
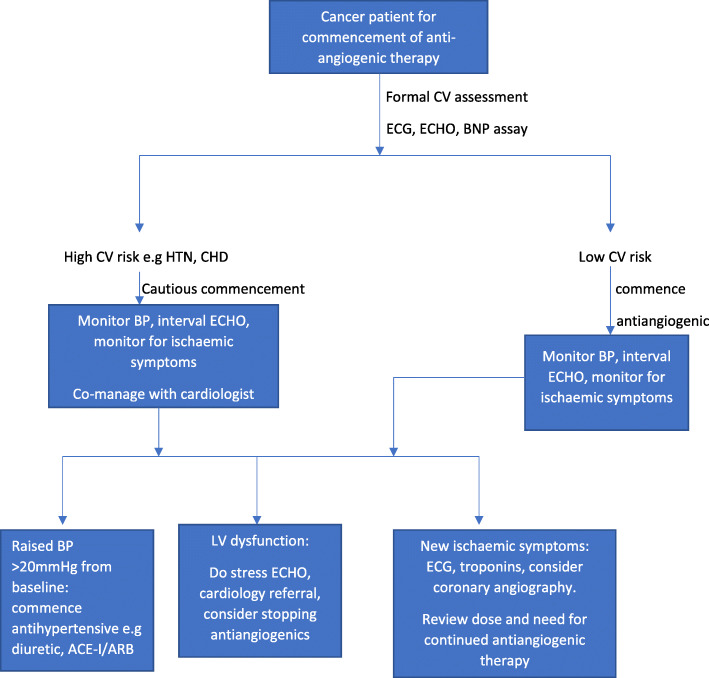
Clinical approach to cardiovascular toxicity of antiangiogenic therapy

The strong link between coronary ischaemia and cardiotoxicity with the use of anti-angiogenic therapy appears to be related to perfusion contraction mismatch [50]. Reduction in nitric oxide signaling and endothelial dysfunction that occur following acute VEGF therapy accelerates coronary vasoconstriction, arterial inflammation, atherosclerosis and platelet reactivity. This is particularly important for those molecules which also affect PDGF signaling where there is decoupling of the pericyte-endothelial myocardial interaction. Theoretical concerns exist for small molecule receptor tyrosine kinase inhibitors about cardiotoxicity and heart failure risk especially in those with pre-existing cardiac diseases due to disruption of AMP-kinase activity [52]. The risk of the left ventricular systolic dysfunction during anti-angiogenic therapy is difficult to predict. Many of the patients in reported studies had been treated with radiotherapy and chemotherapy which may also cause cardiotoxicity. Stress echocardiography may play a role in the evaluation of those with an intermediate or high pre-test probability of coronary artery disease who are being placed on anti-VEGF therapy. Additionally, PET and cardiac MRI may be used to determine myocardial blood flow reserve in these situations. The clinical approach to anti-angiogenic therapy in the setting of cardiovascular risk is presented in Fig. 1.

### Emerging antiangiogenic therapy: nanoparticles and tumour stem cells

Nanoparticles allow absorption of a large quantity of a drug due to the large surface area to volume ratio [53]. Small molecules, proteins, DNA and miRNAs can be loaded into nanoparticles for delivery into tumours. Nanoparticles have advantages over conventional chemotherapy because of their multifunctional targeted roles in the tumour environment. Potential approaches include tissue reoxygenation, either through in situ oxygen supply or increasing intra-tumour hydrogen peroxide metabolism. Organic (liposomes, polymers) and inorganic (gold, silver and silicate) based nanoparticles have been developed for use in experimental tumour models. Nanoparticles of gold, silver, silicate-based and detonated diamond nanoparticles have been shown to inhibit VEGF/VEGFR2 and Akt signaling [54]. Sulphur nanoparticles inactivated the MEK-ERK pathway with consequent inhibition of tumour growth by detaining and binding copper which is an essential co-factor for the MAPK/ERK (MEK) pathway in one study [55].

Some nanoparticles have been designed to silence the expression of HIF-1α gene by antisense oligonucleotides or by miRNAs. Some liposomes carrying camptothecin or topotecan inhibit topoisomerase I [53]. The flow of nanomedicines into tumours may be negatively influenced by hypoxia of tumour microenvironment despite the existence of enhanced permeability and retention effect (EPR) [53]. EPR in solid tumours is due to their vascular abnormalities which lead to extravasation of nanometric molecules in tumours which may thus reach a higher concentration than in normal tissue. The intense hypoxic environment of tumours may be a barrier to the EPR effect. Nanotechnology have circumvented this and can enhance EPRs by using hyperthermia to mediate vascular permeability in solid tumours, ultrasound-induced cavitation to modify tumour tissue, application of nitric oxide-releasing agents to expand blood vessels or administration of antihypertensive to normalize blood flow [53]. These have been achieved in tumours to promote tumour heating using photo-stimulation, magnetism, radiofrequency waves or ultrasound. Tumour vessel normalization has also been attempted using gold nanoparticles to provide human recombinant endostatin (rhEs) in tumours by EPR to facilitate transient vessel normalization and improve anti-tumour therapeutic efficacy. Some have also developed nanoparticles of combination therapy of antiangiogenic and conventional chemotherapy e.g. lipid derivative conjugates (LGCs) containing gemcitabine and paclitaxel to simultaneously restore tumour vasculature and deliver cytotoxic drugs [53]. There is however a need to evaluate the safety and toxicity of nanoparticles. Safety concerns include direct toxicity, nanoparticle aggregate long-term accumulation and immunogenicity. There is also a need to improve drug loading capacity and capability of sustained release of the cargo of nanoparticles in vivo. This will minimize the risk of accumulation of nanoparticles in healthy tissues and facilitate effective delivery to the target tumours. This is important because vascular permeability, oncotic pressure, interstitial pressure and complex nature of tumour stroma affect the movement of nanoparticles in and out of tumour microenvironment. There is a need to stratify patients according to their EPR release to define those patients who can benefit from nanoparticles.

There are different delivery methods for nanoparticles. These include exosomes, plasma membrane coating, use of chitosan and even the use of mesenchymal stem cells. Exosomes allow intracellular delivery of their cargo by fusion of membranes. They can cross biological barriers like the blood-brain barrier easily. Undesired effects of the exosome components and lack of standardized production protocols are limitations to their use. Plasma membrane coating with nanoparticles is another delivery technique for nanoparticles as anti-angiogenics. Examples of nanoparticles delivered this way include tungsten oxide which has been used in lymphoma models [53]. Platelet membranes provide immune evasion and active adhesion to tumour cells due to their P-selectin interaction with ligands expressed on tumour cells. Some have used red cell membranes which are very abundant in the circulation and have immune escape and long circulation time.

Chitosan is another carrier derived from chitin. It is less cytotoxic and is biodegradable and metabolized easily by the kidneys. In mice models of breast cancer, chitosan nanoparticles containing anti-Rho small interfering RNA (siRNA) showed tumour anti-angiogenesis [56]. The binding of αvβ3 integrin to chitosan nanoparticles is an important development. The receptor for αvβ3 integrin is widely expressed in tumours and has shown potentials in ovarian cancer models. Encapsulation of paclitaxel with chitosan nanoparticles has shown efficacy in breast cancer [57]. There is now interest in the use of mesenchymal stem cells (MSCs) to deliver nanoparticles. Hypoxic conditioning of such MSCs used as cell-based therapy can be used for aggressive tumours like glioblastoma multiforme since MSCs can traffic across the blood-brain barrier [53]. Blocking tumour stem cells via anti-angiogenic therapies is another theoretical approach since the tumour stem cell sub-population in some tumours like breast cancers may be more adept at promoting angiogenesis than their non-stem cell counterparts. The different delivery methods for nanoparticles are compared in Table 2.

**Table 2:** Different delivery methods for nanoparticles

Nanoparticle delivery methods	Advantages	Disadvantages
**Exosomes**	Ability to cross natural barriers e.g. blood-brain barrier, autologous use for personalized medicine, provides biocompatibility to nanoparticles	Undesired effects due to the exosome components, lack of standardized production protocols, need to develop techniques for large scale cell culture
**Chitosan**	Less cytotoxic, biodegradable, easily metabolised by the kidneys	
**Plasma membrane coating**	Provides immune evasion, red cell membranes have long circulation time, high versatility, easy fractionalization	Need for high yield methods for membrane derivation, lack of knowledge about all cell membrane components
**Mesenchymal stem cells (MSCs)**	Easy isolation and culture in vitro, non-immunogenicity, tissue regeneration capacity, tumour tropism, migration ability to site of damage, small and relatively homogenous size	Uncertain tumorigenic effect, high retention in the lungs after systemic administration, risk of occlusion of micro-vessels after systemic administration, development of autoantibodies after repeated injections, need for standardized protocols for isolation, purification, and characterization of cell of origin.

## Conclusion

Anti-angiogenic therapy in cancers has enormous potentials using VEGF signaling pathways. Clinical surveillance is important for the early detection of tumour resistance and treatment failure using reliable biomarkers. Cardiovascular toxicity and off-target effects of anti-angiogenic drugs are impediments to their long-term use in those at high cardiovascular risk. Continued research into effective nanoparticle-based delivery methods is an exciting and developing field in cancer therapeutics. It is hoped that the recent interest in mesenchymal cell-based and exosome-based nanoparticle delivery platforms will improve the cellular delivery of newer anti-angiogenics in cancer therapeutics. Understanding of the molecular and cellular mechanisms of tumour angiogenesis will facilitate the development of newer effective anti-angiogenic molecules.

## Data Availability

Not applicable.

## Abbreviations

Ang-1: Angiopoietin 1
bFGF: Basic fibroblast growth factor
CRC: Colorectal cancer
ECs: Endothelial cells
EPCs: Endothelial progenitor cells
EPR: Enhanced permeability and retention effect
FDA: US Food and Drug Administration
FGF: Fibroblast growth factor
GBM: Glioblastoma multiforme
GIST: Gastrointestinal stromal tumour
HCC: Hepatocellular cancer
HGF: Hepatocyte growth factor
HIF-1α: Hypoxia-inducible factor 1-α
LGCs: Lipid derivative conjugates
LV: Left ventricular
MCP-1: Monocyte chemoattractant protein 1
miRNA: MicroRNA
mRCC: Metastatic renal cell cancer
MSC: Mesenchymal stem cell
NSCLC: Non-small cell lung cancer
PD-1: Programmed death 1
PDGF/PDGFR: Platelet-derived growth factor/platelet-derived growth factor receptor
rhE: Recombinant endostatin
RTK: Receptor tyrosine kinase
SDF-1: Stromal cell-derived factor 1
siRNA: Small interfering RNA
TKIs: Tyrosine kinase inhibitors
Tregs: Regulatory T cells
VEGF: Vascular endothelial growth factor
